# Primary care provider perspectives on the role of community pharmacy in colorectal cancer screening: a qualitative study

**DOI:** 10.1186/s12913-023-09828-3

**Published:** 2023-08-23

**Authors:** Alison T. Brenner, Catherine L. Rohweder, Mary Wangen, Dana L. Atkins, Rachel M. Ceballos, Sara Correa, Renée M. Ferrari, Rachel B. Issaka, Annika Ittes, Olufeyisayo O. Odebunmi, Daniel S. Reuland, Austin R. Waters, Stephanie B. Wheeler, Parth D. Shah

**Affiliations:** 1 Division of General Medicine and Clinical Epidemiology, Department of Medicine, University of North Carolina School of Medicine, Chapel Hill, NC US; 2 Lineberger Comprehensive Cancer Center, University of North Carolina at Chapel Hill, Chapel Hill, NC 27599 USA; 3 UNC Center for Health Promotion and Disease Prevention, University of North Carolina at Chapel Hill, Chapel Hill, NC 27599 USA; 4 Hutchinson Institute for Cancer Outcomes Research, Fred Hutchinson Cancer Center, Seattle, WA 98109 USA; 5 Division of Public Health Sciences, Fred Hutchinson Cancer Center, Seattle, WA 98109 USA; 6 Department of Maternal and Child Health, Gillings School of Global Public Health, University of North Carolina at Chapel Hill, Chapel Hill, NC 27599 USA; 7 Division of Gastroenterology, School of Medicine, University of Washington, Seattle, WA 98104, 98109 USA; 8 Department of Health Policy and Management, Gillings School of Global Public Health, University of North Carolina at Chapel Hill, Chapel Hill, NC 27599 USA

**Keywords:** Colorectal Cancer screening, Pharmacy, Fecal immunochemical test, Pharmacist

## Abstract

**Background:**

The United States Preventive Services Task Force (USPSTF) lists 32 grade A or B recommended preventive services for non-pregnant United States (US) adults, including colorectal cancer screening (CRC). Little guidance is given on how to implement these services with consistency and fidelity in primary care. Given limited patient visit time and competing demands, primary care providers (PCPs) tend to prioritize a small subset of these recommendations. Completion rates of some of these services, including CRC screening, are suboptimal. Expanding delivery of preventive services to other healthcare providers, where possible, can improve access and uptake, particularly in medically underserved areas or populations. Fecal immunochemical testing (FIT) (at-home, stool-based testing) for CRC screening can be distributed and resulted without PCP involvement. Pharmacists have long delivered preventive services (e.g., influenza vaccination) and may be a good option for expanding CRC screening delivery using FIT, but it is not clear how PCPs would perceive this expansion.

**Methods:**

We used semi-structured interviews with PCPs in North Carolina and Washington state to assess perceptions and recommendations for a potential pharmacy-based FIT distribution program (PharmFIT™). Transcripts were coded and analyzed using a hybrid inductive-deductive content analysis guided by the Consolidated Framework for Implementation Research (CFIR) to elucidate potential multi-level facilitators of and barriers to implementation of PharmFIT™.

**Results:**

We completed 30 interviews with PCPs in North Carolina (N = 12) and Washington state (N = 18). PCPs in both states were largely accepting of PharmFIT™, with several important considerations. First, PCPs felt that pharmacists should receive appropriate training for identifying patients eligible and due for FIT screening. Second, a clear understanding of responsibility for tracking tests, communication, and, particularly, follow-up of positive test results should be established and followed. Finally, clear electronic workflows should be established for relay of test result information between the pharmacy and the primary care clinic.

**Conclusion:**

If the conditions are met regarding pharmacist training, follow-up for positive FITs, and transfer of documentation, PCPs are likely to support PharmFIT™ as a way for their patients to obtain and complete CRC screening using FIT.

**Supplementary Information:**

The online version contains supplementary material available at 10.1186/s12913-023-09828-3.

## Introduction

The United States Preventive Services Task Force lists thirty-two grade A or B recommendations for non-pregnant adults, including colorectal cancer (CRC) screening [1, 2]. A study recently estimated that delivery of recommended preventive services for a panel of 2500 patients would take approximately 14.1 hours per day on top of acute or chronic disease care and documentation [3]. A recent survey showed that, because of time limitations, primary care providers (PCPs) tend to prioritize 1–3 preventive services per patient per visit [4]. Few adult patients receive all high-priority recommended preventive services, likely due to some combination of time constraints, competing healthcare demands, and infrequent primary care visits [5]. CRC screening rates, in particular, remain suboptimal, with only 67.1% of all United States (US) adults up-to-date with screening in 2019 [6]. People without health insurance or without a regular source of care were screened at a substantially lower rate than the insured or those with regular sources of care [7].

CRC screening using stool-based testing (e.g., fecal immunochemical test (FIT)) can be done by patients at home. Distributing FITs and instructing patients on their use does not require direct PCP input. As such, moving provision of FIT to other healthcare providers is safe and likely to increase appropriate use frequency [8, 9]. Further, expanding access outside of traditional primary care sites has the potential to help address under-screening and screening disparities [10–13].

Community pharmacies may be an ideal option for expanding provision of FITs. Pharmacies have long delivered preventive services such as influenza vaccination. They are the most accessible sources of healthcare services in the United States; patients visit pharmacies two to three times more often than they visit their PCPs [14, 15], and 90% of US residents live within five miles of a pharmacy [16]. Further, a third of pharmacies are located in counties that are rural, low-income, or medically underserved [17]. Community pharmacy practice has increasingly focused on delivery of patient care services over the last 30 years, including preventive care services, and the average community pharmacist today spends approximately 10% of their time providing patient care services not associated with dispensing (e.g., comprehensive medication reviews) [18].

Although the delivery of FIT-based CRC screening share similar workflow processes to other preventive services delivered in pharmacies (like vaccinations), there are notable differences, including that follow-up of a positive FIT result requires colonoscopy, a medical procedure that usually requires a PCP referral. Thus, the routine provision of FIT-based CRC screening in US pharmacies would require buy-in from PCPs who would need to collaborate with pharmacists to develop care coordination processes. However, little is currently known about how PCPs perceive expansion of CRC screening services to community pharmacies [13]. The purpose of this qualitative study was to examine the PCPs’ perspectives of a potential pharmacy-based FIT distribution program (PharmFIT™).

## Methods

### Study design and population

The study was a collaboration between the University of North Carolina at Chapel Hill (UNC) and the Fred Hutchinson Cancer Center in Seattle, WA (Fred Hutch), led by three senior authors (ATB, SBW, PDS). We conducted semi-structured telephone interviews with practicing primary care providers (PCPs) from six public clinics/hospitals serving both rural and urban populations in North Carolina (NC) and Washington (WA).

We (CLR, RMF, PDS, DLA) conducted 30 semi-structured interviews by telephone and Zoom (audio only) with PCPs in NC (n = 12) and WA (n = 18) between August 2019 and January 2020. No interviews were conducted in person. In North Carolina, PCPs were recruited via recommendations from research team members and an advertisement on a department-wide listserv of general internal medicine physicians and residents. In Washington State, interviewees were PCPs who responded to an advertisement on a practice-based research network listserv. The only criterion was that PCPs were currently practicing medicine, and volunteers were scheduled and interviewed in consecutive order until the desired sample size was reached. Given our goal of understanding common perception, among PCPs (a homogenous group), of expanding CRC screening services to pharmacies, we estimated that about 12 interviews per state was sufficient to reach thematic saturation [19, 20]. Participants completed a brief questionnaire (demographic characteristics, current CRC practices) before the interview. Participants were 50% female, 70% White, 23% Asian, and most (83%) had practiced for fewer than six years in internal medicine, family medicine, or adult behavioral and primary care (Table 1).

**Table 1:** Demographic characteristics of PCPs

	All N = 30	North Carolina N = 12	Washington N = 18
Age, mean (SD)	40.7 (9.9)	41.5 (11.5)	40.2 (9.03)
	n(%)	n(%)	n(%)
Female	15 (50)	6 (50)	9 (50)
Physician Role			
Attending	28 (93)	12 (100)	16 (89)
Resident	2 (7)	0 (0)	2 (11)
Specialty			
Internal Medicine	14 (43)	6 (50)	7 (39)
Family Medicine	16 (53)	5 (42)	11 (61)
Behavioral/Primary Care	1 (3)	1 (8)	0 (0)
Years in practice			
Less than 1 year	3 (10)	2 (17)	1 (6)
1–4 years	18 (60)	8 (67)	10 (56)
5–10 years	4 (13)	1 (8)	3 (25)
10 + years	5 (17)	1 (8)	4 (22)
Race			
Non-Hispanic White	21 (70)	12 (100)	9 (50)
Asian	7 (23)	0 (0)	7 (39)
Other/More Than One	2 (7)	0 (0)	2 (11)

### Measures

#### Interview guide

The interview comprised questions informed by the Consolidated Framework for Implementation Research (CFIR), first edition. CFIR is a meta-theoretical framework used to assess determinants of implementation across five domains (intervention characteristics, outer setting, inner setting, characteristics of individuals, and process); it can help researchers assess potential barriers and facilitators in preparation for implementation [21]. There was no previously established relationship between the interviewers and interviewees; participants were given no information about the research study beyond what was presented in the interview guide (described below) and study information sheet. The interview guides were developed by the study team and reviewed by physicians (RBI, DSR) with experience in CRC screening and implementation research. Interviews were conducted by trained study staff at UNC and Fred Hutch (CLR, RMF, PDS, and DLA) via HIPAA-compliant video conferencing software, audio-recorded, and professionally transcribed verbatim. Interviews lasted an average of 31 min (Range: 21–48; NC: 32, 21–48; WA: 29, 19–48).

First, we asked interviewees to describe existing relationships with external pharmacies and pharmacists and how they typically communicate with them. Next, we inquired about their general perceptions of pharmacists providing preventive services, including CRC screening. We also asked about experiences coordinating care with patients’ pharmacists. Next, PCPs were presented with a description of a potential pharmacy-based FIT distribution program (PharmFIT™). PCPs were then asked about the pros and cons of PharmFIT™ and what role they envisioned for themselves. We inquired about potential facilitators and barriers to conducting a PharmFIT™ program, concerns about pharmacists determining patient eligibility for FIT, and opinions regarding standing orders or prescriptions for FIT from PCPs. Respondents were asked to describe how pharmacists might transmit test results back to them, and whether the electronic health record (EHR) would help or hinder communication of results. Further, we assessed how PCPs felt about pharmacists effectively counseling patients on FIT-based screening including test results, and how they envisioned roles and responsibilities for coordinating follow-up care (particularly for positive FIT results). Finally, we asked them how they thought their patients would accept CRC screening from a pharmacy. The interview guide is available as Additional File 1.

### Data analysis

We initially summarized transcripts using a rapid analysis approach [22, 23] to identify themes. Next, we used a hybrid inductive-deductive content analysis approach to interpret transcripts, guided by CFIR constructs from each of the five domains. This allowed for additional standalone codes where new themes were identified [21, 24]. The coding team (CLR, RMF, MW, PDS, and DLA) performed two rounds of consensus coding (one transcript per round), where transcripts were reviewed and coded by all team members to ensure consistent code application. Through this consensus process, we made iterative changes to the codebook by removing inapplicable CFIR constructs and adding stand-alone codes. The final codebook used is available as Additional File 2. After we reached agreement on the codebook, members of the coding team (CLR, RMF, MW PDS, DLA) split the remaining 28 transcripts for independent coding. Once primary coding was completed, the transcripts were swapped and applied codes were reviewed and confirmed by a second member of the team. Disagreements were resolved by consensus among coders and brought to the study principal investigators (ATB, PDS, SBW) for deliberation when necessary. Analyses included repeated reading of transcripts and creating matrices of CFIR constructs as they related to intervention components [25]. Once all transcripts were coded and reviewed, we created code co-occurrence queries and tables to identify constructs most frequently applied to transcript passages. We identified constructs that mapped to four of the CFIR domains: (1) Intervention Characteristics (Relative Advantage, Complexity); (2) Outer Setting (Patient Needs, Cosmopolitanism); (3) Inner Setting (Compatibility); and (4) Characteristics of Individuals (Knowledge and Beliefs around the Intervention). We used ATLAS.ti version 8 (Berlin, DE) for data analysis and management. We edited quotations included in this manuscript for length and clarity.

## Results

All PCPs (n = 30) supported pharmacies providing FIT kits to their patients. Most PCPs offered both stool-based tests (e.g., FIT) and colonoscopy in their clinicals and saw PharmFIT™ as a potential complement to current screening efforts with an added advantage of pharmacies reaching patients who are not up-to-date with their CRC screening and for whom a return visit is not known.

*I think for my practice, we are probably successfully screening a fraction of the folks who are eligible for screening. And so, just having another trusted member of the team providing access to screening is a good thing.*

*Male Family Medicine Physician, Age 50, Washington*

Two general topics emerged in discussions with PCPs about our proposed PharmFIT™ intervention: (1) *pre-implementation considerations* for distributing FITs in pharmacies, and (2) *potential barriers and facilitators* to PharmFIT™ implementation.

### Pre-implementation considerations

Two major themes emerged as pre-implementation considerations that provide context that PCPs felt were critical for achieving buy-in for the PharmFIT™ program.

*Relationships and communication.* First, PCPs highlighted the need for relationships and established lines of communication with community pharmacists. Often, PCPs reported not personally knowing the pharmacists serving their patients and rarely having in-depth conversations with them. Communication tended to be brief, often discussing medication interactions. Many PCPs highlighted poor interoperability between their clinic’s EHR and pharmacy care management software.

*I think the best thing would be if, electronically, our systems communicated so there could be no room for error. If you rely on fax, there’s a potential that the information doesn’t get to the clinic, and then there’s no feedback on whether it got to the clinic or not. I guess telephone communication is an option, but then who are they trying to reach? They often won’t reach the provider because of how busy our schedules are. ​*

*Female Family Medicine Physician, Age 34, Washington*

Several PCPs did, however, note a close relationship with the clinical pharmacists who were embedded at their clinics. The suggestion was that clinical pharmacists could play a role in bridging communication gaps between primary care and pharmacy settings, but PCPs noted that this would not solve interoperability barriers between electronic record systems.

*Workflow ownership and care coordination.* Many PCPs highlighted the critical need for pre-defined workflows and clear ownership of responsibility for steps where potential patient hand-offs could occur. PCPs were concerned that patients would get lost in the process and that the provider, and the pharmacist, would be uncertain about their responsibility to the patient with regards to follow-up care, highlighting a logistical challenge resulting from lack of established and reliable communication pathways described earlier. Without specified and formalized roles for screening, patients could fall through the cracks.

*If the providers are given the result and they’re having to kind of figure out what was told to the patient and kind of pick up the pieces of, you know, what actually happened at the encounter up front – did you know that if it was positive you were probably gonna need a colonoscopy? And that could present some difficulty, just challenges for the provider trying to figure out – okay, like I had this positive result, but I don’t know what they were told.*

*Male Internal Medicine Physician, age 31, North Carolina*

### Barriers and facilitators to Key Components of PharmFIT™ Program

CRC screening with FIT has multiple steps: (1) identifying patients due for screening and eligible for FIT; (2) distributing FITs; (3) ordering FITs; and (4) communicating test results. PCP’s illustrated their perspectives on each of the key step of PharmFIT™, highlighting potential barriers and facilitators for pharmacies to provide this patient care service.

*Step 1: Identifying eligible patients for FIT.* Because FIT screening is recommended only for those at average risk for CRC [1], it is important to ascertain the patient’s personal and family health history. Most often, this includes simple, self-reportable health information such as previous screening and a limited set of comorbidities (e.g., previously diagnosed CRC or pre-cancerous polyps or inflammatory bowel disease). However, in some rarer cases (e.g., Lynch syndrome), a more complete health history could be beneficial. There was some disagreement among PCPs about pharmacists’ capacity to independently identify patients eligible for FIT.

*Step 1 Facilitators.* Most PCPs felt that pharmacists could determine patients’ eligibility for FIT, because it could be accomplished by asking the patient a limited set of screening questions. Some PCPs also suggested referring eligible patients to the pharmacy for FIT distribution, eliminating the need for pharmacists to screen patients for eligibility.

*They’re trained health professionals, so I think knowing the screening guidelines and knowing the frequency and knowing the patient’s history and family history and risk category, I think if they felt comfortable prescribing, then I think that would be fine.*

*Female Family Medicine Physician, Age 34, Washington*

*Step 1 Barriers.* Many PCPs did express concern about pharmacists lacking access to a patient’s full medical history via the PCP’s EHR. Several providers felt that access to the EHR was critical, especially for high-risk patients with conditions that indicate screening with colonoscopy first. Over-screening was also a concern.

*I would have some concerns that when pharmacies don’t have access to the health record. One concern would be patients getting over-tested.*

*Male Primary Care Physician, Age 42, Washington*

*Step 2: Distributing FITs from the pharmacy.* FITs can be distributed without PCP involvement but should be accompanied by education and counseling around CRC screening. PCPs were enthusiastic about the potential to increase access to FIT through distribution at pharmacies.

*Step 2 facilitators.* PCPs were confident that, with training, pharmacists could deliver high quality education and counseling around CRC screening.

*I think if they had… sufficient training, particularly in how to explain the risks and benefits and follow-up expectations to patients with a variety of different health literally levels, I don’t see any reason they couldn’t.*

*Female Primary Care Physician, Age 36, Washington*

*Step 2 barriers.* PCPs did not describe any notable barriers to distributing FITs in pharmacy settings, viewing the distribution of FITs like dispensing medications or medical devices.

*Step 3: Ordering FITs and receiving test results.* FITs can be ordered by pharmacists without a PCP’s prescription and results can be sent electronically or via fax to the provider indicated on the lab requisition form. PCPs shared potential challenges in this step that should be addressed prior to implementation. For care coordination, however, PCPs felt it was critical that test results, particularly positive results, be transmitted back to them.

*Step 3 facilitators.* Most providers were open to receiving results from tests they had not ordered, likening it to other outside orders.

*I suspect the first few times that it happened, I might be a little bit alarmed. But honestly, similar things happen all the time with skilled nursing facilities and home health agencies that have standing orders that we’re not even aware of where urinalyses and other things come back and I didn’t order them. And so, I think I would deal with them the same way.*

*Female Primary Care Physician, Age 36, Washington*

*Step 3 barriers.* Some PCPs expressed concern about the flow of information when receiving FIT results for tests originating from outside orders.

*Assuming that we are talking about existing patients, there is a chart that exists and if we have communicated with the pharmacy about a prescription, there’s established communication there, I don’t know software-wise what, what could happen if there is a positive or a negative result for a FIT test. So I think despite this being 2019, it’s going to be a fax or a phone call, I think.*

*Male Primary Care Physician, Age 51, Washington*

Specifically, PCPs brought up the lack of bi-directional communication between pharmacy- and clinic-based EHRs. Respondents discussed e-prescribing as a potential avenue for communication, but, while the provider is supposed to be notified when the prescription is filled, many felt e-prescriptions were unidirectional.

*I e-prescribe almost all my medications. And I rarely get back any problems with them, unless something needs a prior authorization or something like that. So, I think for the most part, that part seems like it goes well, but it seems also very unidirectional. So I don’t feel like I get much feedback or communication from the outside pharmacist.*

*Female Family Medicine Physician, Age 34, Washington*

Several PCPs noted that most direct communication that did occur happened sporadically by telephone or fax. These communications were largely regarding refill requests or drug contra-indication questions.

Most PCPs reported a lack of relationship with pharmacies in the community, but many expressed an interest in enhancing it, suggesting that more nuanced communication could improve patient care. They discussed substantial exchange of information, in both directions, regarding prescriptions placed and filled. Several PCPs reported some conversation with pharmacists about potential medication interactions, but these exchanges were few and there was little communication on other topics.

*Step 4: Communicating FIT results to patients.* When a FIT result is negative, results are often communicated by letter and reminder to complete another FIT in one year. Generally, PCPs were not concerned about pharmacists delivering notification of negative FIT results, but some did express some reservations about pharmacists communicating positive results to patients. Wrapped up in these reservations was, by association, coordination of follow-up care for patients with positive results, (described earlier).

*Step 4 facilitators.* PCPs were unconcerned about pharmacists delivering negative FIT results to patients.

*I think [communicating negative results] would be fine. I think they could, with minimal training, do it very well.*

*Male Internal Medicine Physician, Age 67, North Carolina*

*Step 4 barriers.* There was some concern about pharmacists’ ability to communicate the subtleties of a positive FIT result and uncertainty about whether pharmacists had the authority to refer a patient to colonoscopy.

*I guess my first reaction is that it’s not a good idea. Because I’m not sure that they would be able to answer all the patient’s questions. I don’t think that they could anticipate every question the patient might have. And then, generally the next step is getting a colonoscopy, which they can’t order. And so, I feel like if you’re going to discuss positive results, you should be able to do the next step.*

*Female Family Medicine Physician, Age 31, North Carolina*

## Discussion

To alleviate overburdened primary care systems, community pharmacies may be promising expansion sites for CRC screening delivery, particularly for medically underserved populations. In this qualitative study of PCPs in NC and WA, we explored perceptions of a pharmacy-based FIT distribution program called PharmFIT™. PCPs found PharmFIT™ acceptable and were enthusiastic about its potential to expand access to CRC screening. Respondents recommended that PharmFIT™ implementation account for a close linkage between PCP and community pharmacies. In implementation planning for PharmFIT™, a critical step for PCP buy-in will be co-developed workflows that define responsibility for the clinic staff, pharmacy staff, and patients at every step, including a clear hand-off of patients with positive FIT results.

### Communication and care coordination

The confluence of ever-increasing preventive services requirements with physician shortages means that, in order to keep up with public demand, some services might need to be moved to other trusted delivery mechanisms [2]. Patients are increasingly accessing preventive services through pharmacies. Preventive services delivery at pharmacies has precedent, with a nearly 30-year history, beginning with expanded authority to deliver vaccinations in 1996 [26]. Further, the COVID-19 pandemic has accelerated this expansion [27, 28] and shifted patient perceptions of community pharmacists to be more integrated members of the medical team [29, 30]. As of 2016, 48 states have authorized pharmacists to enter into Collaborative Practice Agreements [31], a formal arrangement that allows a physician to delegate some patient care services to community pharmacists [32]. With the expanding scope of pharmacy practice, community pharmacists are increasingly integrated into collaborative care models (e.g., patient-centered medical homes (PCMH)) which emphasize preventive services such as CRC screening, [33–37] paving the way for interventions like PharmFIT™. However, PCP endorsement of CRC screening is an important driver of screening uptake [38, 39]. We have observed this in additional PharmFIT™ formative research [40, 41]. As PharmFIT™ is developed and tested, it will be critical to integrate quality improvement and systems science methods, such as process flow diagramming (PFD), for careful integration of workflows and patient hand-offs.

Clear lines of communication between PCPs and pharmacists can support shared workflows. However, the interviewed PCPs cited concerns about reliable and robust communication with pharmacists, with a lack of EHR interoperability central to this concern. Despite progress in improving interoperability across medical and pharmacy patient care management softwares, as well as e-communication capability, this remained a concern. Examples of progress in this area include work over the past 15 years in The Office of the National Coordinator for Health Information Technology (ONC)[42] and the Pharmacy Health Information Technology (PHIT) Collaborative [43]. These two entities have worked towards improving direct exchange of pertinent health information across EHR and pharmacy platforms (dispensing and care management). In particular, the PHIT Collaborative and others have focused on pharmacist access to health information exchanges and EHRs. The Pharmacist eCare Plan Initiative, a national HL7-compliant standard (Health Level 7, a security standard for the electronic transfer of health information)[44], allows pharmacists to exchange care-related information with a wide range of healthcare practitioners [45]. Fueled by a large Center for Medicare and Medicaid Innovation (CMMI) award, the eCare plan has been broadly implemented across pharmacy dispensing software systems with one vendor alone reporting generating and submitting more than 2 million eCare plans. The eCare Plan standard has potential for use as a means of communication between providers, but the full potential will not be realized until EHR platforms in primary care can receive and integrate data transmitted by pharmacies [33, 46–48]. Preparation for PharmFIT™ should include the development of some type of reliable, secure communication and data exchange method, of which the eCare Plan standard is one option. Lower-technology options include verbal communication and fax.

In considering the optimal design for PharmFIT™, it is important to acknowledge, however, that collaborative working relationships between physicians and pharmacists are driven by more than simply policy and technology infrastructure. Kucukarslan and colleagues described drivers in terms of beliefs and attitudes, reporting that overall physician attitude toward collaborating with pharmacists was largely driven by the belief that the collaboration would lead to improvements in patient adherence to medication [46]. There is evidence that the involvement of a pharmacist in the health care team can improve patient outcomes in a variety of health care delivery areas [47, 48]. This suggests that incorporating physician messaging around the potential for improved patient outcomes, in this case, CRC screening completion, will be important in the roll-out of PharmFIT™.

### Feasibility of PharmFIT™

Our interviews suggest that each of the steps of PharmFIT™ could be feasible, and there is some precedent in the literature for successful cancer screening programs in pharmacies. In a systematic review involving non-US studies, Lindsey, et al. (2015), concluded that it was feasible to recruit patients into cancer screening programs in pharmacies, but that the impact on health outcomes was not well-studied [49]. One small US study compared FIT distribution with a screening reminder in the pharmacy [13]. The study showed promise, with most patients accepting of the model and most (59.3%) patients completing a FIT from the pharmacy. Our PharmFIT™ pilot studies, reported elsewhere [50, 51], show promise of a similarly effective intervention. However, there are logistical considerations that need solving, such as how CRC screening tests would be ordered, resulted, and communicated back to the PCP and patient, as well as how to coordinate follow-up care for patients with positive FIT results. PCPs in this qualitative study cited poor bi-directional and or e-communication with community pharmacists as a barrier that could impact these areas.

Despite these challenges, PCPs in our study were largely supportive of expanding CRC screening services to community pharmacies. In fact, apart from the final steps – positive results communications and follow-up – PCPs cited the strong medical training that pharmacists receive as putting them in an ideal position to take up this preventive care service. Porter and colleagues show that team-based care, including programs like PharmFIT™, can alleviate the burden of primary care, reducing the estimated required 26.7 h of work per day to 9.3 h for a standard panel of around 2,500 pateints [3]. As the scope of pharmacy practice grows [18] and reimbursement models shift towards value based care [52], the groundwork is being laid for moving some preventive care services to pharmacists.

### Implications for PharmFIT™ design

**Fig. 1:**
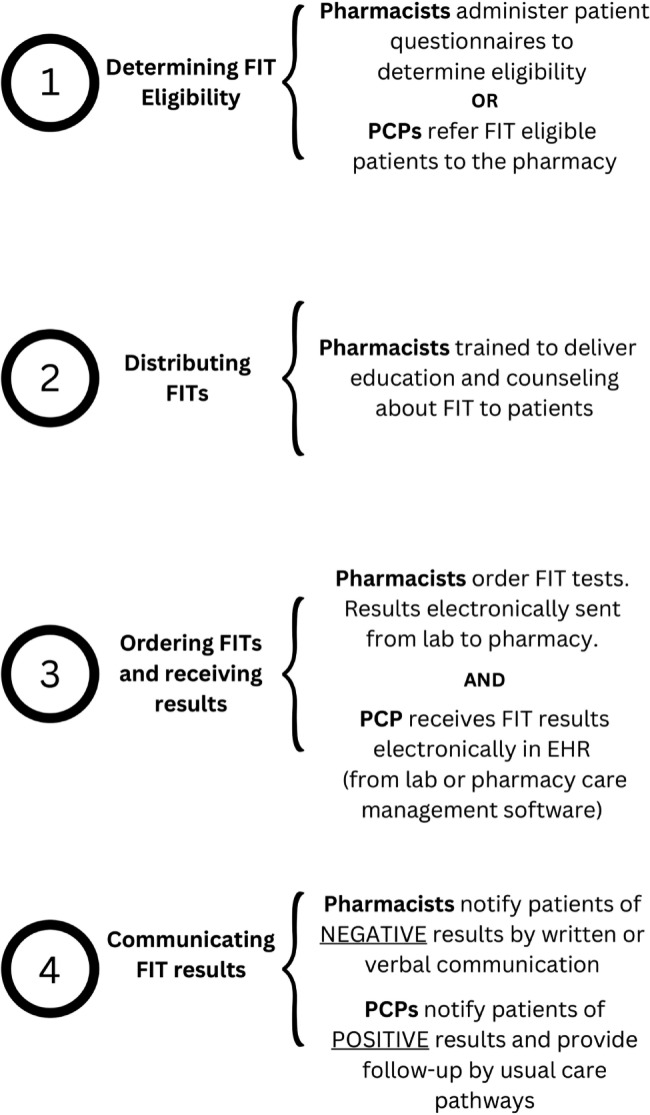
PharmFIT™ design to maximize PCP acceptability

In the analysis of these interviews, essential design elements for each step of PharmFIT™ became clearer (Fig. 1). Many screening services currently performed by pharmacists, such point-of-care tests for viral infections like influenza, result in some type of pharmaceutical intervention. PharmFIT™, however, may be unique in that follow-up, if needed, is another medical service (colonoscopy) that would likely require a PCP referral. Despite this difference, PCPs reported confidence that pharmacists, with appropriate training, can identify patients eligible for FIT and provide appropriate education and counseling. PCPs were also unconcerned about receiving results for a FIT they did not order, but there was some variability in support for pharmacists communicating positive FIT results to patients. In Fig. 1, we depict the specific design for the steps of PharmFIT™, derived from the themes that emerged from these interviews, that may maximize likelihood of PCP buy-in and acceptance of pharmacy-based FIT distribution. Additional challenges, or facilitators, that are unique to PharmFIT™, will become clear in a larger test of this model of cancer screening delivery.

### Strengths and limitations

To our knowledge, this is the first study to directly assess PCPs’ perceptions of a pharmacy-based FIT distribution program called PharmFIT™. Our analysis included PCPs from diverse urban and rural practice settings and multiple primary care disciplines. Additionally, the use of the original CFIR to guide coding and analysis increases the applicability and ease of interpretation of our results. Since our analysis was conducted, a new version of CFIR has been published [53], which could change how we interpret implementation determinants of PharmFIT in future studies. This study also has limitations that should be considered. First, it is possible that additional opinions not reported by our participants could emerge in interviews including PCPs in practice settings not as well represented in our sample (e.g., federally qualified health centers). Additionally, the convenience sample of PCPs represented NC and WA only, states with fairly progressive pharmacy practice policy; it is possible that interviews with PCPs in states with more restrictive practice policies may reveal additional opinions. Further, these interviews were collected prior to the onset of the COVID-19 pandemic and perceptions may have shifted. However, the COVID-19 pandemic has only accelerated the acceptability, growth, and demand of pharmacy-delivered clinical services [28]. It is, thus, unlikely that PCP perceptions of PharmFIT™ have degraded in that time. Finally, while our sample was multi-disciplinary and regionally diverse, we used convenience sampling to recruit participants, potentially biasing results. Future studies should recruit a broader sample of PCPs to determine any variation in themes.

## Conclusions

The results of this formative work suggest that PCPs would be supportive of a pharmacy-based FIT distribution program, given appropriate training of pharmacists, a robust system of PCP-pharmacy communication including EHR interoperability, and clear delineation of responsibility for patient follow-up of test results, particularly positive test results. PharmFIT™ has the potential to significantly expand access to CRC screening. We will report elsewhere patient and pharmacist perceptions of this program. The next steps in this formative work are national surveys with large samples of patients (completed)[40, 54, 55] and pharmacists (underway) to further elucidate the themes captured in the qualitative data interviews. We will be exploring concepts related to acceptability, appropriateness, feasibility, and interest in adopting a PharmFIT™ intervention. We have also completed a pilot of the intervention to assess implementation outcomes and estimate the potential impact on CRC screening rates among adults due for FIT screening who visit community pharmacies [50, 51].

### Electronic supplementary material

Below is the link to the electronic supplementary material.

Supplementary Material 1

Supplementary Material 2

## Data Availability

The dataset supporting the conclusions of this article is available in the UNC Dataverse repository, (https://dataverse.unc.edu/dataverse/cpcrn-4cnc-pharmfit).

## List of abbreviations

CFIR: Consolidated framework for implementation science
CMMI: Center for Medicare and Medicaid Innovation
CRC: Colorectal cancer
EHR: Electronic health record
FIT: Fecal immunochemical test
NC: North Carolina
ONC: Office of the National Coordinator for Health Information Technology
PCMH: Patient centered medical home
PCP: Primary care provider
PHIT: Pharmacy Health Information Technology Collaborative
UNC: University of North Carolina
USPSTF: United States Preventive Services Task Force
WA: Washington
