# Supplementary material for: Primary care provider perspectives on the role of community pharmacy in colorectal cancer screening: a qualitative study
Source: BMC Health Serv Res. 2023 Aug 23;23:892. doi: 10.1186/s12913-023-09828-3 (PMC10463525; doi:10.1186/s12913-023-09828-3)
Supplement: Supplementary file 1 — Supplementary Material 1 [file 12913_2023_9828_MOESM1_ESM.docx]

| **Additional File 1. PharmFIT™ Primary Care Provider Interview Guide** |
| --- |

**Interview Guide for Pharmacy FIT Pilot: Providers**

**Respondents: Primary Care Providers**

Thank you for agreeing to speak with me today for a study being conducted by the Cancer Prevention and Control Network at UNC Chapel Hill. The study involves understanding the acceptance and feasibility of distributing fecal immunochemical test kits or FIT kits in pharmacies. We hope to obtain perspectives concerning FIT kit distribution and to understand what might promote or hinder future implementation of FIT screenings in pharmacies. We are also talking to other primary care providers, pharmacists, and patients.

Before we get started, did you receive the information sheet we sent that has more information about this study? [Review the information sheet with participant]

Do you have any questions before we start the audio-recording?

***START RECORDING AFTER PARTICIPANT HAS READ THE INFORMATION SHEET AND YOU’VE MENTIONED THAT YOU WILL BE AUDIO-RECORDING***

**DEMOGRAPHIC INFORMATION**

1. Can you tell me about the practice where you work?
2. What is your role?
   - What is your title?
3. How long have you worked in this practice?

___less than 1 year

___1-2 years

___3-4 years

___5-6 years

___7-10 years

___10+ years

**SPECIFIC COLORECTAL CANCER PRACTICES**

We’d like to ask you about some of your own practices around colorectal cancer screening.

1. What type of colorectal cancer screenings do you offer to patients?

*[If FOBT/FIT is not offered, ask the following]*

- - What would be your response to a patient requesting a FOBT/FIT kit?

1. What types of kits does your practice provide?
   - Probe: guaiac-based, immunochemical, brand of kit

**PROVIDER’S RELATIONSHIP WITH PHARMACY**

In the next set of questions, we’d like to know a little bit more about what existing relationships you have with pharmacies.

1. What existing relationships do you or your practice have with pharmacies?
   - PROBE: Describe how your practice interacts with specific pharmacies.
   - PROBE: How familiar are you with the pharmacy staff?
   - [Note: Probe about the participants’ own relationships with pharmacies as well as their practice’s relationships.]
2. How does your practice communicate health information to pharmacies?
3. How do pharmacies communicate health information back to your practice?
   - For example, if a patient received a flu shot at a pharmacy, how does the pharmacy send the information back to your practice?
4. How do you feel about pharmacies offering preventive services for patients?
5. What role do you think pharmacies *could* play in distributing self-tests for conditions such as HIV, HPV, yeast infections, and colorectal cancer?
   - What roles *should* pharmacies play?
6. What is your experience coordinating care with patients’ pharmacists?

**PHARMFIT**

Imagine your patients were involved in a pharmacy FIT screening program where patients can get screened directly from pharmacies.

1. How could a program like this be helpful? What might not be helpful?
2. What are the pros and cons of pharmacies providing FIT screenings for your eligible patients?
   - PROBE: What value, if any, might this add for your practice?
3. What role should or could a primary care provider play in a pharmacy-based FIT screening program?
4. If pharmacies were to provide FIT screenings to your eligible patients, what do you see as the barriers/challenges? What about the facilitators?
5. How would you feel about pharmacists determining which patients are eligible for FIT screening?
   - [Note: This question aims to assess participant attitudes about pharmacists assessing patients’ eligibility (e.g. age and comorbidities)]
   - PROBE: How would you feel about a pharmacist distributing kits with or without a standing provider order or prescription?
6. How would you envision pharmacies communicating the results of FIT kits back to your practice?
   - [Note: Ask each of the below questions unless the participant previously provided an answer]
   - How would you feel about a FIT kit result coming to your office from a test that you did not order?
   - How might your electronic medical record system promote or hinder communication about tests results from FIT kits distributed in a pharmacy?
   - What are your concerns, if any, around pharmacies following up with positive test results? Negative test results?
     - PROBE: What do you think about pharmacists discussing positive FIT results with patients?
7. How would you or your practice work with pharmacies to follow-up with patients who completed FIT kits they received from a pharmacy?
   - PROBE: What aspects of follow up do you believe pharmacies should be responsible for?
   - PROBE: What aspects of follow up do you believe you or your practice should be responsible for?
8. How do you think your patients would feel about receiving a FIT kit from a pharmacy?

**CLOSING**

1. Is there anything we did not talk about that you would like to share about colorectal cancer screening though pharmacies?

**DEMOGRAPHIC INFORMATION**

Before we end today, I have a few questions about your background.

1. What is your age? 48
2. Are you…? ___ Male ___ Female male
3. Which one or more of the following would you say is your race?

____White

____Black or African American

____Asian

____Native Hawaiian/Other Pacific Islander

____American Indian or Alaska Native

____Other (Please specify__________________________)

1. What is your ethnicity?

____ Hispanic

____ Non-Hispanic

Thank you so much for participating in this interview. We appreciate your time.
