# Supplementary material for: Primary care provider perspectives on the role of community pharmacy in colorectal cancer screening: a qualitative study
Source: BMC Health Serv Res. 2023 Aug 23;23:892. doi: 10.1186/s12913-023-09828-3 (PMC10463525; doi:10.1186/s12913-023-09828-3)
Supplement: Supplementary file 2 — Supplementary Material 2 [file 12913_2023_9828_MOESM2_ESM.docx]

**Additional File 2. PharmFIT™ Primary Care Provider Interview Codebook**

| **Code** | **Code Definition** | **Code Group** |
| --- | --- | --- |
| **INTERVENTION CHARACTERISTICS** | This code captures how the various key attributes of interventions influence the success of implementation including: adaptability, complexity, cost, design quality/packaging, evidence strength & quality, intervention source, relative advantage and trialability. This domain pertains to the PharmFIT intervention we are trying to develop. | Intervention Characteristics |
| Adaptability | The degree to which an intervention can be adapted, tailored, refined, or reinvented to meet local needs. Examples: using pharmacies for other types of cancer screenings | Intervention Characteristics |
| Complexity | Perceived difficulty of implementation, reflected by duration, scope, radicalness, disruptiveness, centrality, and intricacy and number of steps required to implement. Examples: communication between the PCP clinic and pharmacies; uniformity in patient education; connecting patients to their PCPs or other specialists for colonoscopy needs | Intervention Characteristics |
| Cost | Costs of the intervention and costs associated with implementing the intervention including investment, supply, and opportunity costs. Examples: billing and insurance; cost of training for RPh's or new software for better communication between PCP office and pharmacy | Intervention Characteristics |
| Design Quality/Packaging | Perceived excellence in how the intervention is bundled, presented, and assembled. Examples: education materials for patients, advertisements, etc. | Intervention Characteristics |
| Evidence Strength & Quality | Stakeholders’ perceptions of the quality and validity of evidence supporting the belief that the intervention will have desired outcomes. Example: belief that pharmacist/pharmacy involvement with CRC screening process is beneficial to the patient; Extend to which the PharmFIT/Pharmacy-located CRC screening is evidence-based or evidence-informed | Intervention Characteristics |
| Intervention Source | Perception of key stakeholders about whether the intervention is externally or internally developed. Examples: PharmFIT program was internally developed by the clinic or pharmacy OR externally developed and implemented at the pharmacy/clinic; an existing FIT distrubtion protocol exists/FITs are available at the pharmacy | Intervention Characteristics |
| Relative Advantage | Stakeholders’ perception of the advantage of implementing the intervention versus an alternative solution. Example: Pharmacy-located CRC screening has features that are percieved to improve currently implemented CRC screening programs/processes in primary care clinics | Intervention Characteristics |
| Trialability | The ability to test the intervention on a small scale in the organization, and to be able to reverse course (undo implementation) if warranted. Example: The percieved ability to pilot PharmFIT in collaboration with local pharmacies | Intervention Characteristics |

| **Code** | **Code Definition** | **Code Group** |
| --- | --- | --- |
| **OUTER SETTINGS** | This code captures how to better understand how PCPs feel patient needs, barriers to needs and facilitators to meet those needs, could be accurately addressed by a pharmacy-based FIT screening program. Example: Anything that occurs outside the clinic (working with pharmacies, policies, etc.) | Outer Settings |
| Patient Needs & Resources | The extent to which patient needs, as well as barriers and facilitators to meet those needs, are accurately known and prioritized by the organization. Examples: arranging follow-up care for uninsured/under insured populations; education for low medical-literacy patients; communication with patients | Outer Settings |
| Cosmopolitanism | The degree to which an organization is networked with other external organizations. Examples: the clinic is linked to specialist's in other facilities, cities, etc.; pharmacists reporting back results to PCPs/handoff of care, challenges with not having shared EHR systems between pharmacists and physicians, unidirectional issues | Outer Settings |
| Peer Pressure | Mimetic or competitive pressure to implement an intervention; typically because most or other key peer or competing organizations have already implemented or are in a bid for a competitive edge. Note: Unlikely construct to emerge given current interview questions | Outer Settings |
| External Policies & Incentives | A broad construct that includes external strategies to spread interventions, including policy and regulations (governmental or other central entity), external mandates, recommendations and guidelines, pay-for-performance, collaboratives, and public or benchmark reporting. Examples: Reimbursement concerns (e.g., billing for procedures); Pharamcist accountability to the screening and follow-up processes (training for pharmacists, process for identifying eligible patients, follow-up communication with PCPs and patients; documentation of services; liability); This will also include governing bodies like UNC Hospital/UW Medical Center | Outer Settings |

| **Code** | **Code Definition** | **Code Group** |
| --- | --- | --- |
| **INNER SETTINGS** | This code captures how the structural characteristics, clinic culture, implementation climate and implementation readiness are percieved by PCPs on whether or not an pharmacy-based FIT screening program could be successfully rolled out. Anything pertaining to what is happening in the clinic. | Inner Settings |
| Structural Characteristics | The social architecture, age, maturity, and size of an organization. Examples: how members within a clinic are organized into units; the social hierchy within the clinic. | Inner Settings |
| Networks & Communications | The nature and quality of webs of social networks and the nature and quality of formal and informal communications within an organization. Example: Communication within the clinic (e.g., incoming faxes, how clinicians communicate with other clinicians within the clinic) | Inner Settings |
| Culture | Norms, values, and basic assumptions of a given organization. Example: physician stated an overall mission or objective of their clinic/organization (serving underserved communities, providing holistic care, etc.) | Inner Settings |
| Implementation Climate | The absorptive capacity for change, shared receptivity of involved individuals to an intervention, and the extent to which use of that intervention will be rewarded, supported, and expected within their organization. Examples: physician expressed oppenness to their patients' participation in pharmacy-located CRC screening; clinic is open to a PharmFIT intervention | Inner Settings |
| Tension for Change | The degree to which stakeholders perceive the current situation as intolerable or needing change. Examples: PCP expressed concerns about their current CRC screening practices and recognizes a change needs to occur; PCP expressed rationale for changing their current practice for CRC screening to include community pharmacies. | Inner Settings |
| Compatability | The degree of tangible fit between meaning and values attached to the intervention by involved individuals, how those align with individuals’ own norms, values, and perceived risks and needs, and how the intervention fits with existing workflows and systems. Examples: PharmFIT would not negatively affect current workflow/load within clinic; Perceived fit of PharmFIT within current clinic environment/system | Inner Settings |
| Relative Priority | Individuals’ shared perception of the importance of the implementation within the organization. Example: Where the physician sees changing/improving CRC screening practices in relation to other clinic/physician demands. | Inner Settings |
| Organizational Incentives & Rewards | Extrinsic incentives such as goal-sharing awards, performance reviews, promotions, and raises in salary, and less tangible incentives such as increased stature or respect. Note: Unlikely construct to emerge given current interview questions | Inner Settings |
| Goals & Feedback | The degree to which goals are clearly communicated, acted upon, and fed back to staff, and alignment of that feedback with goals. Note: Unlikely construct to emerge given current interview questions | Inner Settings |
| **Code** | **Code Definition** | **Code Group** |
| Learning Climate | A climate in which: a) leaders express their own fallibility and need for team members’ assistance and input; b) team members feel that they are essential, valued, and knowledgeable partners in the change process; c) individuals feel psychologically safe to try new methods; and d) there is sufficient time and space for reflective thinking and evaluation. Note: Unlikely construct to emerge given current interview questions | Inner Settings |
| Readiness for Implementation | Tangible and immediate indicators of organizational commitment to its decision to implement an intervention. Example: Physician believes their organization would be ready to participate in a PharmFIT intervention | Inner Settings |
| Leadership Engagement | Commitment, involvement, and accountability of leaders and managers with the implementation. Example: Physicians who are in leadership roles in the clinic (e.g. medical director) expressed support or enthusiasm for their clinic to participate in a PharmFIT program; a staff physician perceives their leadership will support PharmFIT for their clinic. | Inner Settings |
| Available Resources | The level of resources dedicated for implementation and on-going operations, including money, training, education, physical space, and time. Example: Personnel and equipment needed to coordinate with pharmacies to conduct CRC screenings and follow-up communication | Inner Settings |
| Access to Knowledge & Information | Ease of access to digestible information and knowledge about the intervention and how to incorporate it into work tasks. Note: Unlikely construct to emerge given current interview questions | Inner Settings |

| **Code** | **Code Definition** | **Code Group** |
| --- | --- | --- |
| **CHARACTERISTICS OF INDIVIDUALS** | This code captures multiple constructs that identify the characteristics of individuals and how this affects program implementation and success including: Knowledge & Beliefs about the intervention; Self-efficacy; Individual Stage of Change; Individual Identification with Organization; and Other Personal Attributes | Characteristics of Individuals |
| Knowledge & Beliefs about the Intervention | Individuals’ attitudes toward and value placed on the intervention as well as familiarity with facts, truths, and principles related to the intervention. Examples: PCP believing this is a good program to implement into pharmacies; Preference for one modality for CRC screening over another; Pharmacists are not capable of screening or counseling patients for CRC | Characteristics of Individuals |
| Self-efficacy | Individual belief in their own capabilities to execute courses of action to achieve implementation goals. Note: Unlikely construct to emerge given current interview questions | Characteristics of Individuals |
| Individual Stage of Change | Characterization of the phase an individual is in, as he or she progresses toward skilled, enthusiastic, and sustained use of the intervention. Note: Unlikely construct to emerge given current interview questions | Characteristics of Individuals |
| Individual Identification with Organization | A broad construct related to how individuals perceive the organization, and their relationship and degree of commitment with that organization. Examples: number of years physician has worked at the clinic; physician's role at the clinic | Characteristics of Individuals |
| Other Personal Attributes | A broad construct to include other personal traits such as tolerance of ambiguity, intellectual ability, motivation, values, competence, capacity, and learning style. Example: A general construct to capture other individual characteristics that have impact the physician's perceptions about PharmFIT not identified by the other constructs | Characteristics of Individuals |

| **Code** | **Code Definition** | **Code Group** |
| --- | --- | --- |
| **PROCESS** | This code captures knowledge building into why implementation succeeds or fails (finding "what works"). This includes: Planning; Engaging; Opinion Leaders; Formally Appointed Internal Implementation Leaders; Champions; External Change Agents; Executing; and Reflecting & Evaluating. This domain focuses on HOW PharmFIT would be implemented by individuals, units, and other stakeholders within and outside the clinic. | Process |
| Planning | The degree to which a scheme or method of behavior and tasks for implementing an intervention are developed in advance, and the quality of those schemes or methods. Example: Physician expresses wanting to co-create PharmFIT protocols with pharmacies | Process |
| Engaging | Attracting and involving appropriate individuals in the implementation and use of the intervention through a combined strategy of social marketing, education, role modeling, training, and other similar activities. Examples: training pharmacists on CRC screening protocols; Communicating with patients about the availability of PharmFIT; Developing communication systems or processes with pharmacies | Process |
| Opinion Leaders | Individuals in an organization who have formal or informal influence on the attitudes and beliefs of their colleagues with respect to implementing the intervention. Example: physician expresses that influential individuals at their clinic would be supportive of clinic's involvement with PharmFIT program | Process |
| Formally Appointed Internal Implementation Leaders | Individuals from within the organization who have been formally appointed with responsibility for implementing an intervention as coordinator, project manager, team leader, or other similar role. Example: Physician identifies who within the clinic (e.g., administrative staff or clinical pharmacist) would collaborate with outside pharmacies to successfully implement PharmFIT for their patients; Person in-charge of QI efforts | Process |
| Champions | “Individuals who dedicate themselves to supporting, marketing, and ‘driving through’ an [implementation]”, overcoming indifference or resistance that the intervention may provoke in an organization. Note: Unlikely construct to emerge given current interview questions | Process |
| External Change Agents | Individuals who are affiliated with an outside entity who formally influence or facilitate intervention decisions in a desirable direction. Note: Unlikely construct to emerge given current interview questions | Process |
| Executing | Carrying out or accomplishing the implementation according to plan. Note: Unlikely construct to emerge given current interview questions | Process |
| Reflecting & Evaluating | Quantitative and qualitative feedback about the progress and quality of implementation accompanied with regular personal and team debriefing about progress and experience. Note: Unlikely construct to emerge given current interview questions | Process |

| **Code** | **Code Definition** | **Code Group** |
| --- | --- | --- |
| **STAND ALONE CODES** | This code captures conversations around ethical considerations, questions and concerns | Standalone Codes |
| Current CRC screening practices | This code captures broadly the physician or clinic's current practices around CRC screening for their patients, including the use of FIT kits or colonoscopy. | Standalone Codes |
| Juicy quote | This code captures important, telling, and striking comments. | Standalone Codes |
| Disagree | This code captures instances when coders disagree with previous code application and will be discussed and reconciled as a gorup | Standalone Codes |
| Electronic health record | Code to capture any times EHRs are mentioned | Standalone Codes |
